# RFX5 in cancer: context-dependent molecular functions and emerging translational relevance

**DOI:** 10.3389/fimmu.2026.1791725

**Published:** 2026-07-01

**Authors:** Li Zhang, Shanmei Du, Kui Liu

**Affiliations:** College of Medical Technology, Zibo Polytechnic University, Zibo, Shandong, China

**Keywords:** biomarker, cancer immunity, context-dependent function, immune checkpoint blockade, PI3K/Akt pathway, RFX5, tumour-intrinsic function

## Abstract

Regulatory factor X5 (RFX5) is a context-dependent transcriptional integrator with key implications for cancer immunotherapy and targeted therapy. In hepatocellular carcinoma (HCC), RFX5 is amplified to drive proliferation and apoptosis resistance via the tyrosine 3-monooxygenase/tryptophan 5-monooxygenase activation protein theta (YWHAQ)–phosphatidylinositol 3-kinase (PI3K)/protein kinase B (Akt) axis. In immune-inflamed tumours, RFX5 regulates antigen presentation, major histocompatibility complex (MHC) class I expression, and CD8^+^ T-cell infiltration, which correlates with enhanced immune surveillance and favourable clinical outcomes. These divergent observations lead us to propose a hypothesis-generating lineage-signal dual-switch framework, which posits that RFX5 functions are dynamically shaped by tumour lineage and microenvironmental immune cues rather than representing fixed oncogenic or tumour-suppressive behaviour. Clinically, altered RFX5 expression correlates with clinical prognosis and immune checkpoint blockade (ICB) response in specific tumour types based on retrospective analyses. Current evidence does not support RFX5 as an independent predictive biomarker, and it may only have reference value when integrated into composite antigen-presentation or MHC signatures. No incremental predictive value beyond established immune biomarkers has been verified. Key translational challenges include defining cell-type-specific targets, distinguishing tumour-intrinsic effects from immune-related alterations, and linking RFX5 activity to therapeutic vulnerabilities.

## Highlights

- RFX5 acts as a context-dependent transcriptional integrator in cancer.
- In HCC, RFX5 drives tumour growth via the YWHAQ–PI3K/Akt axis.
- In immune-inflamed tumours, RFX5 enhances antigen presentation and CD8^+^ T-cell infiltration.
- RFX5 may contribute to composite prognostic and immunotherapy-response signatures in selected immune-inflamed tumours.

## 1 Introduction

### 1.1 The emergence of RFX5 as a cancer-relevant factor

Large-scale pan-cancer genomic and transcriptomic resources, particularly The Cancer Genome Atlas (TCGA), brought RFX5 into view as a cancer-relevant transcription factor with striking tumour-type-dependent expression patterns (1–4). Across malignancies, RFX5 ranges from frequent amplification/overexpression in HCC to modest upregulation with immune-associated correlates in Stomach adenocarcinoma (STAD), and strong linkage to antigen presentation programmes in non-small cell lung cancer (NSCLC) (3, 5, 6). This context-specific behaviour distinguishes RFX5 from canonical oncogenes or tumour suppressors and necessitates lineage- and microenvironment-aware interpretation.

### 1.2 Early observations and clinical associations

Observational studies reported aberrant RFX5 expression in multiple tumour types versus matched normal tissues, suggesting involvement in tumour-associated transcriptional reprogramming (1–3). In HCC, TCGA Liver Hepatocellular Carcinoma (LIHC) analyses have reported RFX5 copy number gain in approximately one quarter of cases, together with increased tumour mRNA expression and nuclear protein overexpression in paired specimens (5, 7). In breast cancer, RFX5 has been reported to associate with clinicopathological features in some cohorts, though interpretation is complicated by immune-contexture confounding and limited large-cohort validation (4, 8). In NSCLC, RFX5 has been incorporated into Antigen Processing Machinery (APM) signatures showing tentative correlations with favourable outcomes and ICB response in retrospective cohorts (6). Notably, its incremental predictive value relative to established biomarkers remains unvalidated. Collectively, these data support recurrent dysregulation and clinical relevance of RFX5, while foreshadowing divergent functional meanings across tumour contexts (2, 3, 6, 8).

### 1.3 Core questions and scope

Although RFX5 has attracted growing research attention, three fundamental questions still await clear answers: (i) What drives the diverse functions of RFX5, including genomic aberrations, TME signalling, chromatin landscapes and their crosstalk? (ii) What are the tumour- and cell-type-specific target genes and coupled signalling pathways of RFX5? (iii) How can RFX5 be utilized as a biomarker or therapeutic target for precision oncology?

To address these conflicting findings and outstanding questions, we propose a hypothesis-generating and experimentally testable lineage-signal dual-switch conceptual framework to reconcile currently conflicting published observations regarding RFX5’s paradoxical roles in cancer. In oncogenic lineages, RFX5 activates the YWHAQ-PI3K/Akt cascade to promote tumour growth; in immune-inflamed tumours, it interacts with NLR family CARD domain containing 5 (NLRC5) to boost MHC-I-driven immune surveillance. This dual regulatory feature distinguishes RFX5 from conventional oncogenes and tumour suppressors (5, 9–11).

In this review, we comprehensively summarize published data on RFX5 expression profiles, biological roles, molecular mechanisms and clinical relevance, with a primary focus on solid malignancies and immunotherapy-related research.

Currently, most related reviews focus on three distinct directions: the general properties of the Regulatory Factor X (RFX) transcription factor family, the conserved regulatory mechanisms of APM, and immune interference in pan-cancer biomarker studies. Rarely have previous studies specifically focused on RFX5 and resolved its paradoxical oncogenic and immunomodulatory functions across different cancer types.

In light of the above limitations, our work delivers three major advances. First, the newly proposed lineage-signal dual-switch conceptual framework reconciles the inconsistent observations of RFX5 activity. Second, we rigorously disentangle tumour lineage effects from microenvironmental immune cues to interpret its functional diversity. Third, we evaluate the clinical value of RFX5 as a prognostic and immunotherapeutic biomarker based on tumour-specific contexts. This work fills critical gaps in current literature and provides novel insights for follow-up studies in RFX5 biology and cancer immunology.

### 1.4 Literature search and selection

This narrative review systematically summarizes the research progress of RFX5 in human malignancies. Standard literature retrieval and screening strategies were applied to prevent citation bias. We primarily searched PubMed, and supplemented our literature retrieval with Web of Science, Embase and Cistrome. The combined search keywords included RFX5, regulatory factor X 5, RFX transcription factor family, tumour, cancer immunity, the PI3K/Akt pathway, phosphatase orphan 1 (PHOSPHO1) and v-myc avian myelocytomatosis viral oncogene homolog (MYC) signalling. We initially retrieved 201 relevant publications from PubMed. The earliest available literature on RFX5 dates back to 1995.

We included peer-reviewed English full-text studies focusing on human RFX5, including original research, clinical analyses and omics studies. Conference abstracts, case reports, non-English articles, duplicate papers and irrelevant studies were excluded. During sorting, we categorized literature by tumour types and research directions. Functional experiments, ChIP-seq analyses, multi-omics research and large clinical cohorts were prioritized. Conflicting results were interpreted comprehensively in combination with tumour characteristics and immune microenvironments to ensure objective discussion.

## 2 The multifaceted and context-dependent role of RFX5 in cancer: molecular mechanisms and clinical implications

**Figure 1:**
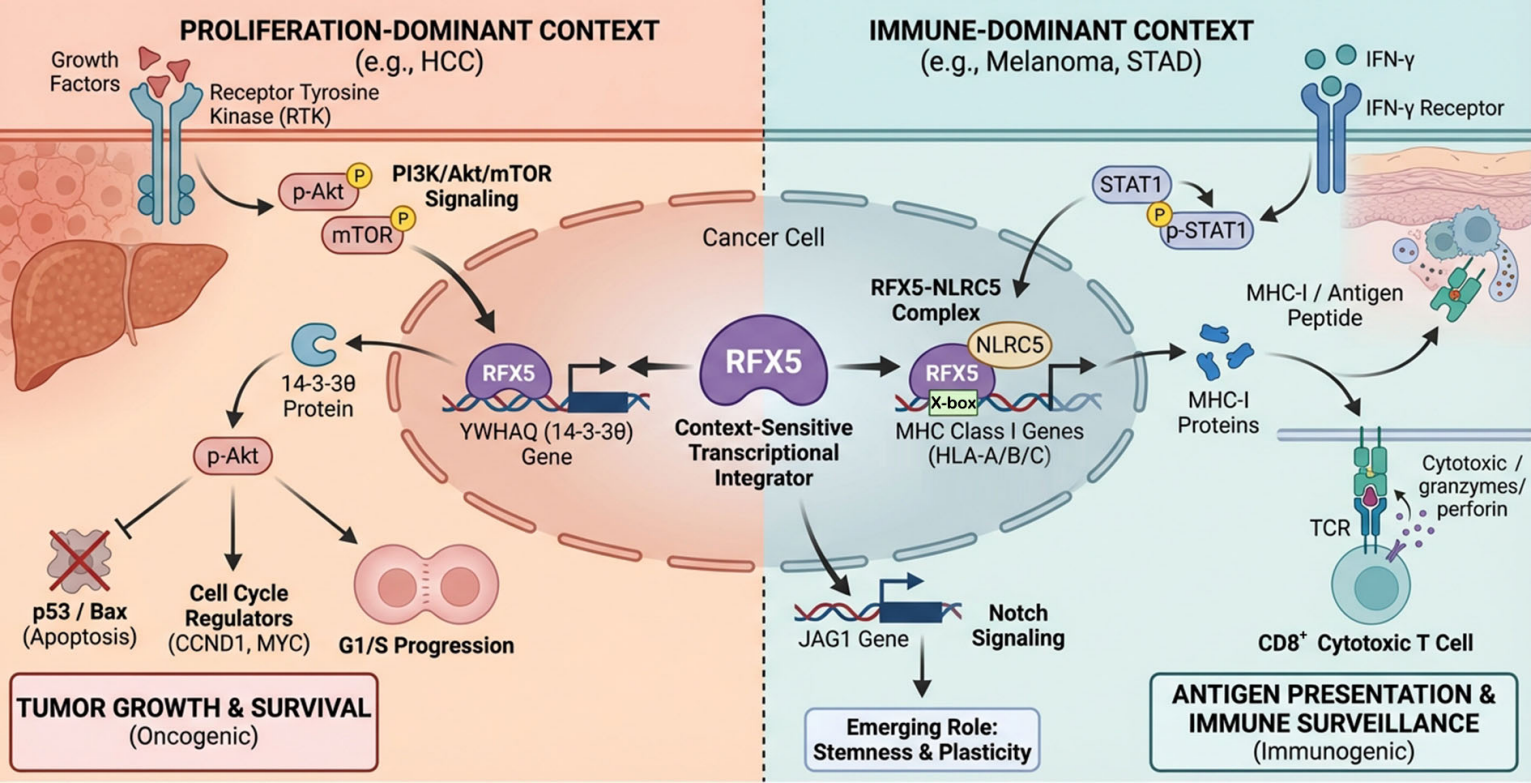
Context-dependent functional heterogeneity of RFX5 in cancer. RFX5 functions as a context-sensitive transcriptional integrator whose outputs are dictated by tumour lineage and dominant signaling cues. In proliferation-driven contexts, such as HCC, RFX5 activates the YWHAQ-PI3K/Akt axis to facilitate tumour growth and survival; in immune-inflamed malignancies including NSCLC, STAD and breast cancer, RFX5 interacts with NLRC5 to boost MHC-I-driven immune surveillance. Its associations with tumour cell plasticity and stemness remain largely correlative and require further experimental validation.

Notably, given the limitations of bulk RNA-seq, it remains challenging to definitively distinguish RFX5 expression originating from tumour cells versus immune or stromal cells. For most cancer types except HCC, the observed associations between RFX5 and clinical or molecular phenotypes should be interpreted as correlative rather than causal. Cancer-type-specific expression patterns, biological features, and clinical relevance of RFX5 are summarized in **Table 1**. To clearly present the uneven evidence across different cancer types, we further graded available evidence systematically, as summarized in **Table 2**. Pan-cancer transcriptomic analyses integrating TCGA and Genotype-Tissue Expression (GTEx) datasets consistently demonstrate that RFX5 expression is highly tumour-type dependent, with substantial variability across solid malignancies. Importantly, bulk tumour RNA-seq data integrate tumour-cell-intrinsic transcription with variable immune and stromal contributions (12, 13). Deconvolution analyses using tools such as the Tumour Immune Estimation Resource (TIMER) and Cell-type Identification By Estimating Relative Subsets of RNA Transcripts (CIBERSORT) frequently report positive correlations between RFX5 expression and inferred cytotoxic immune infiltration, particularly in immune-inflamed tumour types (3, 12). The divergent functional programmes of RFX5 across proliferation-dominant and immune-dominant tumour contexts are illustrated in **Figure 1** to support our testable conceptual framework. This schematic diagram illustrates our core lineage-signal dual-switch conceptual framework: in oncogenic proliferation-driven tumours (represented by HCC), RFX5 activates the YWHAQ-PI3K/Akt axis to facilitate tumour growth and survival; in immune-inflamed malignancies, RFX5 interacts with NLRC5 to boost MHC-I-mediated antigen presentation and anti-tumour immune surveillance. Collectively, this figure summarizes the core mechanism of the lineage-signal dual-switch conceptual framework proposed in this review. A simplified, streamlined version of this model is provided in **Supplementary Figure 1**.

**Table 1:** Cancer-type-specific expression patterns and clinical relevance of RFX5.

Cancer type	RFX5 expression pattern	Major associated biological features	Clinical or prognostic relevance	Representative evidence
HCC	Amplified and overexpressed	Promotes proliferation, inhibits apoptosis through transcriptional activation of YWHAQ	High RFX5 expression correlates with worse clinical outcomes and mediates oncogenic activity	TCGA-LIHC + functional tests
Breast cancer	Variable across subtypes	Associated with immune signatures	No confirmed links to stage/metastasis	TCGA-BRCA analyses
NSCLC	Heterogeneous expression	Immune-related, not a core driver	Prognostic role unproven; part of APM signatures	Transcriptomic studies
STAD	Moderate upregulation	Dominantly linked to immune profiles	High expression correlates with better survival	TCGA-STAD datasets

**Table 2:** Evidence grading of RFX5 across cancer types.

Cancer type	Tumour-intrinsic mechanistic evidence	Bulk cohort prognostic evidence	Immune deconvolution & correlative analysis	ICB predictive evidence	Single-cell/spatial evidence
HCC	Strong (well-validated)	Strong	Moderate	Limited	Limited
Breast cancer	Absent	Moderate	Strong	Limited	Limited
NSCLC	Absent	Moderate	Strong	Moderate (retrospective)	Limited
STAD	Absent	Strong	Strong	Limited	Limited

Single-cell and spatial transcriptomic studies have revealed pronounced intratumoural immune heterogeneity across solid tumours, yet dedicated mechanistic analyses for RFX5 remain scarce, restricting causal inference on its cellular origin and function (14–16).

### 2.1 HCC

HCC represents the most well-established context for tumour-intrinsic RFX5 function (5, 17). Genomic profiling of the TCGA-LIHC cohort reveals recurrent RFX5 copy number gain, which correlates strongly with increased tumour mRNA and protein expression relative to adjacent non-tumour tissues (5, 7). Immunohistochemical analyses of tissue microarrays further confirm nuclear RFX5 overexpression in a majority of HCC specimens, with notable intratumoural heterogeneity (5).

Functional studies provide compelling causal evidence: short hairpin (shRNA)- or clustered regularly interspaced short palindromic repeats/CRISPR-associated protein 9 (CRISPR/Cas9)-mediated depletion of RFX5 suppresses proliferation, clonogenic growth, and xenograft tumour formation, whereas ectopic expression enhances these phenotypes (5, 17). Mechanistically, RFX5 directly binds the promoter of YWHAQ, transcriptionally activating its expression, as validated by chromatin immunoprecipitation and luciferase reporter assays (18). Rescue experiments demonstrate that YWHAQ overexpression reverses the growth inhibition induced by RFX5 knockdown, establishing the RFX5-YWHAQ axis as a critical pro-tumourigenic pathway. This axis promotes tumour cell survival by attenuating p53–Bax-mediated apoptosis and engaging Akt-associated signalling (5, 19). Beyond YWHAQ, RFX5 transcriptionally activates multiple non-MHC downstream targets, including tripeptidyl peptidase 1 (TPP1), lysine demethylase 4A (KDM4A), and SCL/TAL1 interrupting locus (STIL). These targets mediate distinct oncogenic pathways, including cell-cycle progression, apoptosis resistance, metabolic reprogramming, and stemness-associated phenotypes (5, 7, 17, 20–22).

Clinically, elevated RFX5 expression consistently associates with poor clinical outcomes in HCC, reflecting its tumour-intrinsic oncogenic role (5). High expression of RFX5 downstream targets also correlates with unfavourable prognosis across independent cohorts (5, 7, 17). Collectively, these data support RFX5 as a transcriptional driver of HCC progression, with potential therapeutic relevance in this specific tumour context (5, 17).

### 2.2 Breast cancer

In TCGA Breast Invasive Carcinoma (BRCA), RFX5 expression exhibits at most modest and often non-significant elevation relative to normal tissue, and large-cohort validation remains limited (8, 23). Mechanistically, RFX5-dependent regulation of the Notch ligand Jagged 1 (JAG1) has been reported in triple-negative breast cancer (TNBC), suggesting a potential link to plasticity-associated transcriptional programmes (4). However, definitive functional validation of stemness or tumour-initiating capacity remains insufficient.

Clinically, reported prognostic associations of RFX5 in breast cancer are inconsistent across studies. Available evidence indicates that RFX5 expression in breast tumours is closely linked to immune-associated transcriptional programmes, including antigen presentation and cytotoxic T-cell–related pathways, rather than reflecting tumour-cell-intrinsic oncogenic activity (8, 24). To date, no robust large-cohort studies have demonstrated a consistent, independent prognostic value of RFX5 in breast cancer after adjustment for immune infiltration and clinicopathological variables. Although immune infiltration and immune-related gene expression are known to vary substantially across breast cancer molecular subtypes, direct subtype-stratified analyses specifically interrogating RFX5 expression and function remain limited (25). Accordingly, current data indicate that altered RFX5 expression in breast cancer correlates strongly with immune-related transcriptional programmes. The observed prognostic associations are derived from bulk dataset analyses and may largely reflect tumour immune contexture, rather than tumour-cell-intrinsic oncogenic activity. At present, RFX5 cannot be regarded as a validated independent prognostic biomarker for breast cancer. Rigorous subtype stratification, immune-adjusted clinical analyses and dedicated functional assays are needed to verify these hypotheses and further clarify the clinical relevance of RFX5 in breast cancer.

### 2.3 NSCLC

NSCLC is characterized by prominent immune heterogeneity across patients. Diverse immunosuppressive tumour microenvironments within tumours are major contributors to resistance against ICB therapy (26, 27). Bulk transcriptomic analyses of TCGA Lung Adenocarcinoma (LUAD) and TCGA Lung Squamous Cell Carcinoma (LUSC) reveal that RFX5 expression correlates with MHC-related genes, interferon-γ-responsive signatures, and cytotoxic immune infiltration (6). Such immunological variation is largely driven by defective antigen presentation, which is a key driver of ICB resistance (6). Correlative analyses indicate that RFX5 expression is linked to antigen processing and presentation programmes. Composite APM signatures incorporating RFX5 show statistical associations with improved ICB response in retrospective cohorts (6). However, this correlation remains exploratory. No multivariable regression or treatment-interaction analyses have verified that RFX adds incremental predictive value over established biomarkers such as programmed death-ligand 1 (PD-L1), tumour mutational burden (TMB) and interferon-gamma (IFN-γ) signatures. Thus, RFX5 is not suitable for use as a standalone predictive biomarker for ICB treatment. Few studies have stratified NSCLC patients by histological subtypes, and causal functional validation is still lacking. Although transcriptional signatures based on antigen processing and presentation machinery correlate with ICB outcomes in NSCLC, most relevant studies are retrospective and lack functional and mechanistic validation of RFX5 (6). Existing transcriptomic studies have revealed extensive immune heterogeneity across NSCLC tumours. Nevertheless, the distinct roles of RFX5 in diverse immune phenotypes have not been fully characterized, and its reliability as an independent biomarker still requires further verification (6).

Overall, RFX5 expression in NSCLC should be regarded as a context-related immune-associated marker that reflects tumour immune visibility and antigen presentation. Its apparent clinical correlations require further verification via large-scale prospective cohorts and multivariable-adjusted analyses that account for immune confounding factors.

### 2.4 STAD

Integrative analyses of TCGA, GTEx, and independent validation cohorts demonstrate that RFX5 mRNA is significantly upregulated in STAD tissues compared with adjacent normal gastric tissues (3). Genomic variation of RFX5 in STAD occurs at a moderate frequency, predominantly comprising missense mutations and C>T single nucleotide variants, while copy number variation is reported without consistent evidence of focal amplification across cohorts (3).

Functionally, STAD differs markedly from HCC. RFX5 expression shows strong positive correlations with immune-related pathways—including T-cell activation and antigen receptor-mediated signalling—and the abundance of CD8^+^ T cells, CD4^+^ T cells, and myeloid dendritic cells (3, 28). These immune associations are further contextualised by pan-cancer immune subtype analyses, which indicate that gastrointestinal cancers are enriched in IFN-γ-dominant (C2) immune-inflamed and inflammatory (C3) subtypes, with C3 associated with favourable pan-cancer survival (1). Functional enrichment analyses further implicate RFX5-associated genes in MHC complex binding, antigen receptor-mediated signalling, and Th17 cell differentiation (3). Notably, no studies have directly examined the epigenetic regulation of RFX5 or its downstream targets in STAD, representing an important mechanistic gap (3).

In bulk cohort analyses, higher RFX5 expression correlates with favourable overall survival, first progression, and post-progression survival (3). Multivariate Cox regression analyses confirmed that RFX5, together with pathological stage, is an independent prognostic factor, yet this correlation cannot differentiate the respective contributions of tumour cells and immune cells (29). These associations are further supported by qRT-PCR validation confirming RFX5 upregulation at the mRNA level in STAD tissues (3). The favourable prognostic association of RFX5 in STAD reflects an immune-inflamed tumour microenvironment rather than a tumour-suppressive role of RFX5 in gastric epithelial cells. Accordingly, RFX5 should not be interpreted as a standalone tumour-intrinsic prognostic marker in STAD without immune-adjusted modelling.

### 2.5 Functional roles across cancer hallmarks: tumour-intrinsic versus immune-mediated outputs

At the core of RFX-driven tumour-intrinsic malignancy lies its pro-proliferative and cell cycle-regulatory activity. In triple-negative breast cancer models, RFX5 depletion inhibits cancer cell proliferation, while RFX5 overexpression enhances the malignant cellular phenotypes (17). RFX5 expression varies greatly across human malignancies, and its dysregulation is closely linked to the transcriptional networks controlling cell proliferation and cell cycle progression. Mechanistically, RFX5 exerts its oncogenic effects via transactivating downstream oncogenes. This regulatory mechanism has been fully verified in HCC cell models, where TPP1 is confirmed as a direct downstream target of RFX5 (7). Notably, the pro-proliferative effect of RFX5 is most pronounced in HCC cases characterized by amplification-driven RFX5 overexpression (7, 23). However, outside the context of HCC, direct experimental validation of the causal mechanistic links underlying RFX5-mediated tumour promotion remains limited.

Beyond proliferation, RFX5 facilitates tumour cell migration and invasion, with its dysregulated expression being closely associated with epithelial–mesenchymal transition (EMT)-related transcriptional alterations and cytoskeletal reorganization (4). Collectively, in models of HCC, breast cancer, and NSCLC, RFX5 depletion consistently impairs the migratory and invasive capabilities of tumour cells (4, 30). Although direct regulation of canonical EMT master regulators has not been experimentally demonstrated, RFX5 appears to modulate invasion-associated transcriptional programmes rather than triggering a complete epithelial–mesenchymal transition (4, 31). This pro-invasive function exhibits tumour-type specificity: the most pronounced phenotypes are observed in human epidermal growth factor receptor 2 (HER2)-positive and basal-like breast cancer subtypes, as well as in HCC cases with RFX5 amplification. For instance, in breast cancer, although direct subtype-specific studies on RFX5 are limited, evidence from triple-negative/basal-like models shows that RFX5 overexpression enhances cell migration and invasion, consistent with stronger phenotypes in non-luminal, aggressive subtypes (e.g., basal-like). In HCC, RFX5 is frequently overexpressed, and its elevated expression correlates with tumour progression, suggesting that RFX5 amplification or overexpression may contribute to aggressive HCC biology (4). Outside of these well-characterized tumour contexts, direct functional validation of RFX5-mediated regulation of migration and invasion remains limited.

RFX5 also exerts context-dependent control over tumour cell apoptosis and survival signalling—two additional hallmarks that synergize with migration and invasion to drive tumour progression and metastasis. In HCC, RFX5 promotes tumour cell survival by modulating the p53 pathway: RFX5 depletion increases cell apoptosis, whereas RFX5 overexpression reduces apoptotic rates (17, 20). In breast cancer, RFX5 confers strong pro-survival and pro-invasive capabilities on basal-like tumour cells. The oncogenic activities of RFX5 are closely linked to this aggressive subtype (23). In NSCLC, the survival-related functions of RFX5 are closely linked to cell-cycle progression (30). By contrast, in STAD, high RFX5 expression is linked to better clinical outcomes and extensive immune infiltration, while its core function is not dominated by intrinsic cell survival pathways (3). Collectively, these observations indicate that RFX5-mediated biological functions vary across distinct tumour genetic backgrounds and microenvironments.

Complementing these core malignant traits, RFX5 is implicated in tumour cell stemness and plasticity, predominantly through correlative transcriptomic analyses. Pan-cancer transcriptomic analyses reveal that RFX5 expression correlates with stemness indices in breast and lung cancers. Specifically, in triple-negative breast cancer, RFX5 directly transactivates JAG1, activating Notch signalling and promoting proliferation, migration, and resistance to apoptosis—traits consistent with cancer stem cell-like properties (2, 4). Nevertheless, direct causal evidence for RFX5 in core stemness properties, such as self-renewal and tumour-initiating capacity, remains limited. Gold-standard functional assays—such as sphere formation, limiting dilution transplantation, and lineage tracing—are needed to definitively establish RFX5’s mechanistic contributions to core stemness properties.

In parallel to its tumour-intrinsic functions, RFX5 acts as a conserved regulator of tumour immune visibility through transcriptional activation of MHC class I genes. As a core component of the RFX complex, it cooperates with NLRC5 to induce MHC-I expression and antigen-processing machinery, including low molecular mass polypeptide 2 (LMP2), LMP7, and transporter associated with antigen processing 1 (TAP1) (10, 32, 33). Across pan-cancer cohorts, RFX5-high tumours display elevated MHC-I expression, enhanced antigen-presentation programmes, and increased CD8^+^ T-cell signatures in breast cancer, NSCLC, and STAD (2, 3, 8). Given that RFX5 regulates MHC-I expression and antigen-presentation pathways, its expression level may indirectly correlate with the clinical response to immune checkpoint inhibitors across several tumour types. These findings are purely exploratory. To date, there is no evidence proving RFX5 can act as an independent predictive biomarker or deliver additional predictive value compared with widely used immune-related signatures. Conversely, as a key component of the RF complex required for NLRC5-mediated MHC-I transcription, loss or silencing of RFX5 diminishes MHC-I expression and facilitates immune escape in various cancers (34).

### 2.6 A hypothesis-generating conceptual framework: context-dependent dual functions of RFX5

Collectively, available evidence indicates that RFX5 is neither a fixed oncogene nor a constitutive tumour suppressor. Instead, it functions as a context-sensitive transcriptional integrator whose outputs are determined by tumour lineage, dominant signalling cues, and the cellular composition of the tumour microenvironment.

In growth-factor-dominant contexts, exemplified by HCC, RFX5 couples to proliferation and survival programmes through direct transcriptional targets such as YWHAQ (5). In contrast, in immune-inflamed contexts, including subsets of NSCLC, STAD and breast cancer, RFX5 aligns with IFN-γ-associated antigen-presentation programmes and enhanced cytotoxic immune infiltration (3, 6, 8, 23, 30).

In STAD, multiple analyses consistently show that high RFX5 expression tracks with increased cytotoxic immune infiltration and favourable clinical outcomes (3). During tumour progression, gastric cancer frequently exhibits increased angiogenesis-related activity, including vascular endothelial growth factor (VEGF)-vascular endothelial growth factor receptor (VEGFR) signalling, which is relevant to late-stage biology and therapeutic response. Although direct mechanistic links between RFX5 and angiogenic transcription have not been established, co-expression analyses suggest that RFX5-associated functional programmes may shift as the tumour microenvironment evolves. This represents a plausible but hypothetical, and experimentally testable, model for stage-dependent functional diversification. The above context-dependent regulatory patterns are synthesized and hypothetically summarized in **Figure 1** as a testable conceptual model.

## 3 Molecular mechanisms and clinical translation of context-dependent RFX5 function

The functional heterogeneity of RFX5 across cancers arises from context-specific regulatory networks and dynamic interactions with tumour signalling pathways and chromatin landscapes. Elucidating these molecular mechanisms, together with their translational implications and limitations, is essential for evaluating RFX5 as a precision oncology biomarker.

### 3.1 Molecular mechanisms underlying context-dependent RFX5 function

#### 3.1.1 Canonical DNA binding and promoter logic

RFX5 is a conserved member of the RFX transcription factor family, defined by sequence-specific recognition of X-box motifs in target promoters (35–37). Its N-terminus mediates dimerization and assembly with Regulatory Factor X-Associated Protein (RFXAP) and Regulatory Factor X-Associated Ankyrin-Containing Protein (RFXANK) into the functional trimeric RFX complex, whereas its C-terminus facilitates cooperative binding with NF-Y to stabilize promoter occupancy (38).

The most established biological function of RFX5 lies in immune gene regulation. The RFX5-RFXAP-RFXANK complex is essential for MHC-II enhanceosome assembly and class II major histocompatibility complex transactivator (CIITA)-dependent transcription in professional antigen-presenting cells (39–41). In parallel, RFX5 cooperates with NLRC5 to regulate MHC-I expression and antigen-processing genes such as LMP2 and TAP1, forming a core regulatory axis for both MHC classes (10). Consistent with these roles, RFX5-high immune-inflamed tumours exhibit enhanced antigen presentation and cytotoxic immune infiltration, including increased expression of MHC-I/II molecules, CD8^+^ T-cell markers, and Th1/Th17-associated pathways (3, 8).

Tumour-intrinsic RFX5 targets are highly context-dependent and have been fully validated in HCC, where RFX5 is frequently amplified. Chromatin immunoprecipitation (ChIP) and luciferase assays demonstrate direct RFX5 binding to the YWHAQ promoter, activating PI3K/Akt signalling to promote proliferation and G1/S transition. RFX5 also upregulates KDM4A, repressing p53-p21 signalling and further facilitating cell-cycle progression (3). In triple-negative breast cancer, RFX5 directly activates JAG1, driving Notch signalling in a subtype-specific manner (4).

Outside HCC and selected TNBC models, direct causal evidence for a conserved tumour-intrinsic growth programme remains limited. This likely reflects context-dependent promoter accessibility, lineage-specific cofactors, and tumour-specific genetic alterations, such as amplification in HCC versus epigenetic regulation in other cancers. Overall, RFX5 target selection and functional output are tightly constrained by cellular and microenvironmental context.

#### 3.1.2 Transcriptional cooperation with signalling programmes and chromatin context

RFX5 transcriptional activity is shaped by context-specific signalling inputs and chromatin accessibility (42–44). Notably, RFX5 binding is influenced by local chromatin state, and its occupancy is modulated by promoter/enhancer accessibility and the presence of cofactors, enabling engagement with regions that may be transcriptionally silent under other conditions. This property allows RFX5 to regulate target genes in a context-dependent manner.

In immune-responsive settings, IFN-γ signalling is a dominant upstream regulator of antigen-presentation programmes. RFX5 is required for both basal and cytokine-inducible MHC-I transcription. IFN-γ/signal transducer and activator of transcription 1 (STAT1) signalling establishes a permissive chromatin state at antigen-presentation loci, facilitating assembly of the RFX5-NLRC5 transcriptional complex (10, 45). Although direct STAT1-RFX complex interactions have not been conclusively demonstrated, functional convergence is supported by coordinated induction of MHC-I, TAP1, and LMP2 following IFN-γ stimulation or RFX5 overexpression (46). Some early biochemical evidence suggests that RFX family proteins, including RFX5, are capable of interacting with histone deacetylase 2 (HDAC2) *in vitro*, indicating potential context-dependent interactions with chromatin modifiers (47).

In growth-dominant contexts such as HCC, RFX5 is incorporated into oncogenic transcriptional networks that prioritise cell-cycle progression and survival. Direct activation of YWHAQ enhances Akt phosphorylation, suppresses p53-dependent apoptosis, and accelerates G1/S transition. This effect is reinforced by KDM4A-mediated repression of p53-p21 signalling and is amplified by genomic RFX5 overexpression in HCC. In this setting, RFX5 chromatin occupancy is biased toward growth-associated genes, likely driven by oncogenic signalling-induced accessibility changes and context-specific cofactors.

Together, these findings support a model of contextual coupling rather than tumour-agnostic oncogenicity, whereby the same DNA-binding factor produces distinct hallmark outputs depending on signalling milieu and chromatin landscape. RFX5’s binding flexibility and cooperative interactions enable adaptive regulation of transcriptional programmes across diverse tumour contexts.

### 3.2 Clinical translation of RFX5 function

#### 3.2.1 Translational considerations and limitations

At present, RFX5 is best regarded as a biological indicator of tumour transcriptional state rather than a validated therapeutic target, reflecting its strong association with immune contexture in bulk transcriptomic analyses rather than consistent tumour-intrinsic oncogenic activity (1). Direct pharmacological targeting is challenging because RFX5 is a nuclear transcription factor with essential roles in antigen presentation, and indiscriminate inhibition may impair anti-tumour immune responses (46, 48). Therefore, translational strategies are more credible when aimed at context-specific modulation of RFX5-associated pathway dependencies or at preserving/restoring immune visibility programmes in immune-evasive tumours, rather than directly targeting RFX5 itself.

Key limitations for clinical deployment include: (i) cell-of-origin attribution (tumour vs immune/stromal source of bulk signal) (49, 50); (ii) separation of predictive versus prognostic effects via treatment-interaction testing; (iii) confounding by IFN-γ signalling in immune-inflamed tumours (51); and (iv) assay standardisation and reproducible cut-offs across platforms, as widely discussed in immune-oncology biomarker studies.

#### 3.2.2 RFX5 as a biomarker related to immunotherapy response

**Figure 2:**
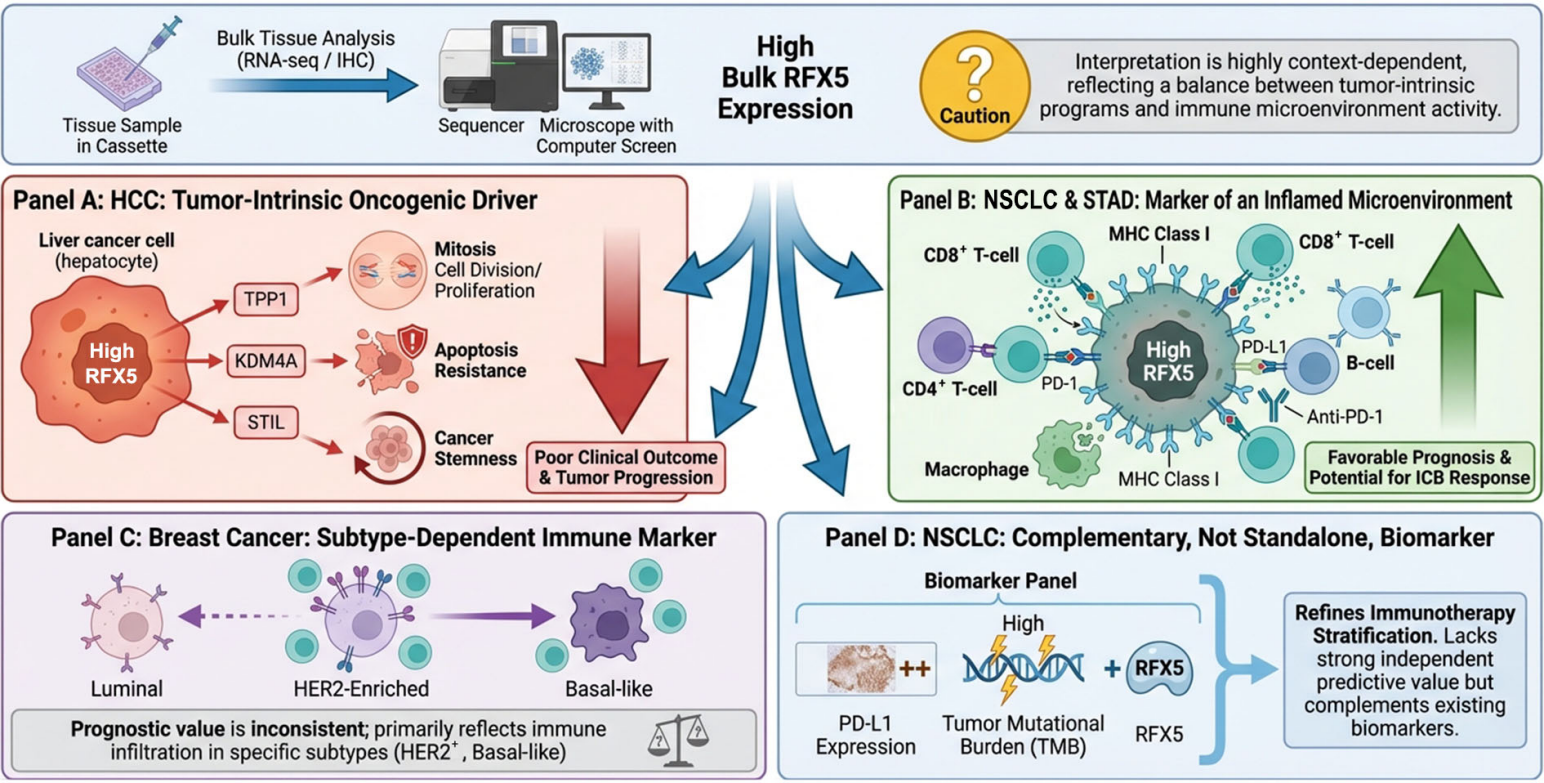
Context-dependent biological and clinical implications of high bulk RFX5 expression across solid tumours. **(A)** illustrates the oncogenic, cell-intrinsic functions of RFX5 in HCC. Elevated RFX5 transcriptionally activates TPP1, KDM4A and STIL to promote cell proliferation, apoptosis resistance and cancer stemness, driving tumour progression and inferior patient survival. **(B)** represents typical immune-inflamed malignancies such as NSCLC and STAD [breast cancer immune signatures are separately summarized in **(C)**], where elevated RFX5 correlates with potent anti-tumour immunity and favourable treatment response. RFX5 cooperates with NLRC5 to maintain robust MHC class I presentation, facilitating CD8^+^ T cell recruitment and potentiating ICB efficacy. Panel C summarizes subtype-specific RFX5 patterns in breast cancer. RFX5 prognostic relevance is weak in luminal subtypes, while its high expression marks abundant anti-tumour immune infiltration in HER2-enriched and basal-like tumours, leading to inconsistent overall predictive value across all breast cancer patients. **(D)** demonstrates that RFX5 acts as a complementary rather than standalone predictive biomarker for immunotherapy in NSCLC. Combined with PD-L1 expression and TMB, RFX5 refines patient stratification without delivering independent predictive performance on its own.

The predictive relevance of RFX5 for immunotherapy response is highly context-dependent and reflects the balance between tumour-intrinsic transcriptional programmes and immune microenvironment activity (**Figure 2**). **Figure 2** further stratifies the clinical implications of RFX5 across different tumour types and molecular subtypes. Each subpanel corresponds to a typical cancer scenario: it distinguishes proliferation-dominant tumours, immune-inflamed tumours and breast cancer subtypes, and elaborates the varied prognostic and predictive values of RFX5 in each context. Specifically, **Figure 2A** illustrates the profile of proliferation-dominant HCC, where high RFX5 exerts intrinsic oncogenic effects. **Figure 2B** represents typical immune-inflamed malignancies such as NSCLC and STAD, where elevated RFX5 correlates with potent anti-tumour immunity and favourable treatment response. **Figure 2C** focuses on breast cancer and highlights the subtype-dependent characteristics of RFX5. **Figure 2D** demonstrates that RFX5 serves as a complementary biomarker within multi-gene signatures for immunotherapy stratification in multiple solid tumours. Overall, rather than acting as a universal predictive biomarker, RFX5 appears to contribute to immunotherapy responsiveness in a lineage- and context-specific manner.

In immune-inflamed tumour settings, including subsets of NSCLC and STAD, high RFX5 expression is tightly coupled to activation of antigen processing and presentation machinery, elevated MHC class I expression, and increased infiltration of cytotoxic immune cells (10, 34, 46). Transcriptomic analyses of checkpoint inhibitor-treated cohorts indicate that antigen-presentation signatures incorporating RFX5 correlate with improved progression-free and overall survival in retrospective cohorts, reflecting an interferon-responsive and immune-active tumour microenvironment (**Figures 2B, D**).

It is worth noting that RFX5 cannot serve as an independent ICB predictive biomarker. Current evidence fails to verify its incremental value over classic immune indicators, and it is only suitable for inclusion in composite antigen-presentation or MHC-related biomarker panels for clinical interpretation (**Figure 2**).

By contrast, in proliferation-dominant tumours such as HCC, high RFX5 expression predominantly reflects tumour-intrinsic oncogenic functions, including promotion of cell-cycle progression, apoptosis resistance, and stemness-related programmes via pathways such as YWHAQ-PI3K/Akt and KDM4A-mediated p53 suppression (5). These tumours frequently exhibit limited immune infiltration and are generally less responsive to ICB monotherapy, indicating that elevated RFX5 in this setting does not confer immunological vulnerability but instead tracks with aggressive tumour biology (**Figure 2A**). Notably, direct clinical evidence linking RFX5 expression alone to immunotherapy outcomes in HCC remains lacking, underscoring the need for cautious interpretation.

In breast cancer, the clinical significance of RFX5 as an immunotherapy-related biomarker is further modulated by molecular subtype (4, 8, 23, 30). In HER2-enriched and basal-like tumours, RFX5 expression correlates with immune infiltration and antigen-presentation activity, whereas in luminal subtypes its prognostic and predictive value is inconsistent, reflecting weaker immune engagement and greater dependence on hormone-driven programmes (**Figure 2C**).

## 4 Future perspectives: unresolved questions and emerging directions

### 4.1 Priority biological gaps

A central unresolved issue is whether clinically relevant RFX5 expression derives from tumour cells, immune cells, or both; single-cell and spatial profiling across stages and treatment contexts is required to resolve cellular origin and niche-specific regulatory patterns (52, 53). Outside HCC, context-specific direct targets remain incompletely defined; integrating RFX5 CUT&RUN/CUT&Tag with ATAC-seq will be essential to distinguish direct regulation from co-expression with IFN-driven programmes. Upstream regulators of RFX5 have not been fully elucidated, which likely explains why RFX5 is driven by copy number variation (CNV) in HCC but modulated by immune cues in other tumours (1, 3, 7). Finally, associations with plasticity/stemness signatures are intriguing but not causal; rigorous state-transition and functional self-renewal assays with rescue experiments are needed to clarify driver versus marker roles (54).

To experimentally validate the lineage-signal dual-switch conceptual framework and disentangle compartment-specific RFX5 functions, multiple targeted study designs are proposed. First, combining single-cell RNA-seq with spatial transcriptomics can spatially map RFX5 expression across tumour parenchyma and immune niches, distinguishing transcriptomic signals derived from tumour cells versus immune cells. Second, compartment-resolved CUT&RUN/CUT&Tag assays, performed separately in sorted tumour cells and immune cells, identifying compartment-specific target genes and regulatory circuits. Third, integrating spatial proteomics or cytokine detection to map IFN-γ gradients will help clarify how inflammatory microenvironmental signals redirect RFX5 transcriptional activity toward antigen presentation programmes in immune-inflamed regions. These multi-omics and spatial approaches will solidify the core arguments of our proposed model. Consistent with the above evidence stratification, follow-up functional assays are required to verify the conclusions in breast cancer, NSCLC and STAD, and clarify cell-type-specific roles of RFX across diverse tumour microenvironments.

### 4.2 A biomarker-focused validation roadmap

To explore its potential clinical value within composite signatures, future studies should prioritise: (i) prospective cohorts with harmonised endpoints and pre-specified thresholds; (ii) multivariable models adjusting for PD-L1, TMB, IFN-γ programmes, and immune infiltration to quantify RFX5’s incremental predictive value (ΔC-index/ΔAUC) (55); (iii) treatment-interaction analyses distinguishing predictive versus prognostic effects; (iv) cross-platform concordance testing to establish reproducible cut-offs; (v) cell-type-resolved validation to dissect RFX5 signals from tumour or immune compartments (49). Spatial metabolomic profiling in HCC reveals that Treg-specific metabolic rewiring remodels the immunomodulatory function of transcription factors including RFX5, while systematic multi-omics consensus frameworks standardise cross-cohort, liquid biopsy and radiomics biomarker validation pipelines (56, 57). Collectively, these advances confirm single-gene molecules fail to act as independent ICB predictors and require multi-layer, cell-type-specific verification to achieve reliable clinical stratification (58–60).

### 4.3 Translational opportunities: programme-level modulation over direct targeting

Given RFX5’s essential immune functions and limited druggability, direct inhibition is unlikely to be broadly safe or beneficial (46, 48). More realistic strategies include targeting context-specific pathway dependencies in RFX5-high tumours (e.g., PI3K/Akt in HCC), and restoring or maintaining antigen presentation/immune visibility programmes in immune-evasive states using upstream modulators where appropriate (5, 34). Such approaches naturally align with programme-level patient stratification to rationalise immunotherapy combinations.

## 5 Conclusion

This review proposes a hypothesis-generating lineage-signal dual-switch framework to interpret the dual roles of RFX5 across solid tumours. Well-established findings are limited to HCC: RFX amplification activates the YWHAQ-PI3K/Akt axis to drive tumour proliferation and apoptosis resistance, which is supported by rigorous *in vitro* and *in vivo* functional assays. In HCC, elevated RFX5 expression reliably correlates with poor patient survival.

Most observations in breast cancer, NSCLC and STAD remain inferential. These results are derived solely from bulk transcriptomic and clinical correlative analyses. RFX5 expression in these immune-inflamed tumours is closely linked to MHC-I-mediated antigen presentation and CD8^+^ T cell infiltration, and correlates with favourable prognosis and ICB response. However, bulk data cannot separate RFX5 signals derived from tumour cells versus immune or stromal compartments.

For clinical translation, several critical validations are still required: cell-type-resolved experiments to define cellular sources of RFX5 expression, prospective cohorts and multivariable analyses to confirm its independent and incremental predictive value beyond PD-L1, TMB and other established immune biomarkers, and standardized detection protocols for clinical application.

Given current evidence, RFX5 is not reliable as a standalone prognostic or ICB predictive biomarker. It is more appropriate to incorporate RFX5 into composite immune or APM signature panels for tumour stratification. Additionally, direct pharmacological targeting of RFX5 is not feasible due to its essential physiological function in antigen presentation; targeting its downstream pathways represents a more viable therapeutic strategy.

## Supplementary material

Supplementary Figure 1 Simplified single-panel schematic of the RFX5 lineage-signal dual-switch conceptual framework. This streamlined diagram illustrates the core context-dependent functions of RFX5. Left section: In HCC, RFX5 mediates the oncogenic programme by transcriptionally activating YWHAQ and subsequently triggering the PI3K/Akt pathway, which promotes tumour proliferation and anti-apoptosis. Right section: In immune-inflamed malignancies, RFX5 acts in concert with NLRC5 to elevate MHC class I expression and facilitate antigen presentation, thereby increasing CD8^+^ T cell infiltration and strengthening anti-tumour immunity.
