# Supplementary figures and images for: RFX5 in cancer: context-dependent molecular functions and emerging translational relevance
Source: Front Immunol. 2026 Jul 1;17:1791725. doi: 10.3389/fimmu.2026.1791725 (PMC13368746; doi:10.3389/fimmu.2026.1791725)

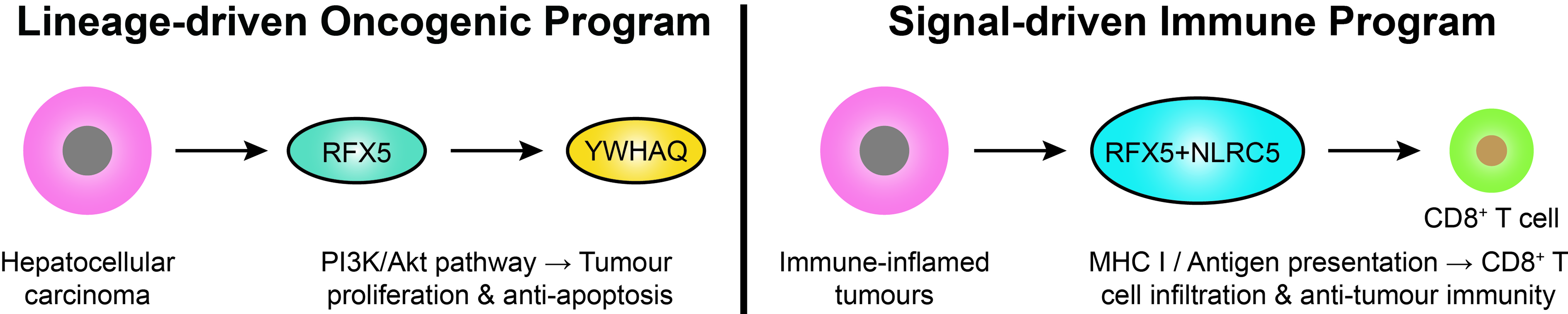

Supplement: Supplementary Figure 1 — Simplified single-panel schematic of the RFX5 lineage-signal dual-switch conceptual framework. This streamlined diagram illustrates the core context-dependent functions of RFX5. Left section: In HCC, RFX5 mediates the oncogenic programme by transcriptionally activating YWHAQ and subsequently triggering the PI3K/Akt pathway, which promotes tumour proliferation and anti-apoptosis. Right section: In immune-inflamed malignancies, RFX5 acts in concert with NLRC5 to elevate MHC class I expression and facilitate antigen presentation, thereby increasing CD8+ T cell infiltration and strengthening anti-tumour immunity. [file Image1.tif]
